# Analysis of angiogenic markers in oral squamous cell carcinoma-gene and protein expression

**DOI:** 10.1186/s13005-015-0076-7

**Published:** 2015-06-05

**Authors:** Susanne Jung, Sonja Sielker, Nikolai Purcz, Christoph Sproll, Yahya Acil, Johannes Kleinheinz

**Affiliations:** Vascular Biology of Oral Structures (VABOS) Research Unit, Department of Cranio-Maxillofacial Surgery, University Hospital Muenster, Waldeyerstraße 30, D-48149 Muenster, Germany; Department of Cranio-Maxillofacial Surgery, University Hospital Kiel, Kiel, Germany; Department of Cranio-Maxillofacial Surgery, University Hospital Duesseldorf, Duesseldorf, Germany

**Keywords:** Oral squamous cell carcinoma, Microarray analysis, Angiopoietins, VEGF, EFNB2, Angiogenesis

## Abstract

**Purpose:**

Therapeutic strategies attacking oral squamous cell carcinoma have not essentially succeeded to improve long-term prognosis and overall survival over the last decades. Therefore, in this study, we aimed to illuminate the molecular regulation of angiogenesis in this tumour entity in order to demask novel markers of prognosis or therapeutic approach.

**Materials and methods:**

A panel of significant transcriptional alterations in angiogenic genes of 83 cancer samples was established by comparison to 30 samples of healthy oral mucosa with microarray technique. Immunohistochemistry (IHC) was performed to trace the signalling cascade from gene to protein level.

**Results:**

A distinctive expression profile of VEGFA, EFNB2, PECAM1/CD31, ANGPT1 and ANGPT2 was revealed: VEGFA, EFNB2, and ANGPT2 were found overexpressed in 84 % to 95 % of tumour samples. In contrast, the expression of CD31 and ANGPT1 was downregulated in 80 % to 95 % of tumour samples. IHC confirmed results of the microarray analysis.

Tumours with lymphatic spread showed higher gene expression rates of VEGFA, EFNB2 and ANGPT2 in moderately differentiated tumours and of VEGFA and EFNB2 in small tumours, respectively. The ANGPT1/ ANGPT2 transcription ratio was found decreased in larger tumours and especially in tumours without lymphatic spread.

**Conclusions:**

A characteristic expression profile of angiogenic markers was established. The specific overexpression of EFNB2 in small tumours with lymphatic spread and the typical decrease of the ANGPT1/ ANGPT2 ratio in larger tumours give weight to EFNB2 and angiopoietins as prognostic factors and potential therapeutic targets.

## Introduction

Oral squamous cell carcinoma (OSCC) is one of the most commonly diagnosed cancer entities in the world and is associated with unchanged high morbidity and mortality. Nearly 500 000 cases are diagnosed every year, and over 250 000 patients find death due to the disease [1, 2].

Tracing current experimental and clinical results, growth and progression of the disease are closely related to a functioning vascularisation. As in other malignant tumours, an increasing vascularity, from healthy mucosa over dysplastic lesion to invasive carcinoma has been observed [3, 4]. With regard to this, different angiogenic effects are exerted by the vascular endothelial growth factor (VEGF). It promotes endothelial cell growth as well as proliferation and it induces vascular permeability allowing for cell migration. Analysis of VEGF gene expression in OSCC revealed an obvious up-regulation of the mitogen in the majority of studied tumour samples which correlated with tumour size [5].

Kaemmerer et al. were able to show that higher microvessel density was associated not only with higher tumour stage as well as earlier relapse, but also with a higher rate of metastasis and significantly decreased overall and disease-free survival [6]. These findings provide the base for and strongly suggest further elucidation of VEGF expression and regulation in malignant tissues.

As a further factor, Angiopoietin 1 (ANGPT1) plays a significant role in endothelial cell survival and vascular maturation. Its molecular action leads to a tightening of cell junctions and reduces cell permeability and inflammatory response. In colorectal cancer, reverse effects of ANGPT1 have been detected: ANGPT1 overexpression in combination with VEGF seems to induce angiogenesis whereas ANGPT1 alone seems to exert an anti-angiogenic effect by stabilizing the existing vasculature, and thus reducing any remodelling effects [7]. Abnormal levels of ANGPT1 and Angiopoietin 2 (ANGPT2), together with their receptor, have been observed in prostate and breast cancer [8].

ANGPT2, in general, acts as ANGPT1-Tie antagonist and exerts anti-angiogenic effects in several tumour entities. In OSCC, ANGPT2 overexpression comes along with increased malignancy and poor prognosis [9]. In other cases, ANGPT2 signalling is able to induce blood vessel degradation and endothelial cell sensitization following the impact of angiogenic cytokines such as VEGF; moreover, ANGPT2 induces apoptosis in endothelial cells in the absence of VEGF. ANGPT2, as well as VEGF are ligands for a receptor specific tyrosine kinase that is expressed on endothelial cells (ECs), exclusively. This ligand-receptor interaction results in vessel maturation and growth [9]. In addition, ANGPT2 plays a significant role during early stages of the angiogenic switch in the formation of tumours when it induces apoptosis in endothelial cells; this results in massive hypoxia in the concerned tumour tissue. As a consequence, overexpression of VEGF and the reestablishment of a viable vasculature arises [10]. The synergistic effect of VEGF and ANGPT2 then obviously results in tumour angiogenesis and poor prognosis. Therefore, an increased expression of ANGPT1 and ANGPT2 in tumour tissue hints to an escalation of tumour malignancy on the grounds of an altered vascularisation.

Ephrin B2 (EFNB2) is a member of the receptor protein-tyrosin kinase family and is involved in many developmental processes; all members of the ephrin-B family are transmembrane proteins. First observations led to the assumption that a stronger expression of EFNB2 is associated with an increased vascularisation and tumour growth as observed in human colorectal cancer. Afterward closer investigation, the newly formed vessels were found functionally inadequate and tumour growth showed a marked decrease [11]. Due to their critical position in the angiogenic pathway, ephrins turned out as operative therapeutic targets: intervention with selective EFNB2 antibodies resulted in impaired HUVEC migration and maturation, altered tube formation, and in reduced angiogenesis [12].

The aim of this investigation was to examine the expression of key regulators of the angiogenic and vasculogenic cascades in OSCC tumour samples, from the stimulation via VEGF to the mature endothelial cells marked by CD31. Crucial questions were: (i) Is there a significant expression profile? (ii) Are the selected angio- and vasculogenic markers expressed? (iii) Does an altered expression correlate firstly with an immunohistochemical detection of the protein, and secondly with clinical parameters such as survival, metastasis or recurrent disease?

## Material and methods

### Patient data

For this retrospective analysis, 83 tissue samples were taken during tumour surgery after informed consent of the patients in the years 2009–2012. Included were patients with a histological diagnosed squamous cell carcinoma of the oral cavity. These patients were over 18 years old and had not received any adjuvant radiation or chemotherapy. Patients with recurrent disease were included.

Healthy tissue controls (n = 30) were taken from oral vestibular mucosa samples during orthognathic or traumatologic surgery after informed consent. The tissue samples were snap-frozen in liquid nitrogen after surgery and stored at −80 °C until further usage. The Ethics Committee of the medical faculty approved the study setup; the ID of the ethical clearance (WWU Muenster) is 2008-580-f-s, and the study is registered in a public Clinical Trials Registry, DRKS00000199.

### RNA extraction and microarray assay

Total RNA was prepared by Qiazol extraction and purification with the miRNeasy Mini Kit (Qiagen). Purity and integrity of the isolated total RNA was assessed on the Agilent 2100 bioanalyzer (Agilent Technologies). For microarray analysis, we used the Agilent Array platform employing the manufacturer’s standard protocols for sample preparation and microarray hybridization. Gene expression analysis was performed with the Whole Human Gene Expression Microarray (4x44K; GPL4133; Agilent Technologies). After the washing steps arrays were scanned using the Agilent G2505B Microarray Scanner (Agilent Technologies) and feature extraction was performed with Feature Extraction software version 9.5 (Agilent Technologies). Data files from mRNA microarrays were analysed by GeneSpring GX 7.3.1 according to manufacturer’s protocol (Agilent Technologies). The first normalisation step consisted of background elimination while in a second step the 50^th^ percentile of each spot was normalized. Normalisation to healthy oral mucosa pool was performed in the last step with the expression factor for the healthy oral mucosa pool set to 1. Primary statistical analysis was performed with GeneSpring GX 7.3.1 software.

### Immunohistochemistry

A selection of 14 paraffin embedded tumours samples providing a representative amount of tissue for immunohistochemical staining was analysed in this investigation. IHC was performed after histopathological confirmation of OSCC. After examination only 14 of the 83 tissue samples provided enough tumour tissue to allow for a decent analysis, these were included in the study.

Primary antibodies applied in this work: VEGF Ab-3 (clone JH12; NeoMarkers, Germany), CD31/PECAM1 (clone JC70A; dilution 1:20; Dako, Germany), Ephrin B2 (Abcam, United Kingdom), Angiopoietin 1 (clone N-18) and Angiopoietin 2 (clone F-1; Santa Cruz, CA, USA). The immunohistochemical procedure was performed with the Dako REAL™ Detection Kit for VEGF Ab-3 and the Dako LSAB™+ System-AP for Ephrin B2, Angiopoietin 1, and 2 according to the manufacturer’s protocols (Dako, Germany). For primary antibody detection of CD31, the Dako EnVision™ + System- HRP was used in combination with the Dako AEC+ High Sensitivity Substrate Chromogen (Dako, Germany) according to the manufacturer’s protocols.

Negative as well as positive controls were implemented according to manufacturer’s protocols. Staining results were summarized in an immunoreactive score (IRS) based on the multiplication of percentage of positive cells (PP) and staining intensity (SI) ranging from 0 to 12. Definition of PP score: PP = 0 no staining, PP = 1 staining in less than 10 % of cells, PP = 2 staining in 10 to 50 % of cells, PP = 3 staining in 50 to 80 % of cells, and PP = 4 staining in more than 80 % of cells. Definition of SI score: SI = 1 no staining, SI = 2 weak staining, SI = 3 moderate staining, and SI = strong staining.

### Statistical analysis

Statistical analysis of expression factors and IRS score according to tumour size, UICC stages and grading was carried out by one way ANOVA using a modified Levene testing and *p* < 0.05, and a PostHoc analysis with Bonferroni-Holm testing (Daniel’s XL Toolbox version 6.53; http://xltoolbox.sourceforge.net).

## Results

### Patient data

Tissue samples of 83 patients were analysed. Patients age ranged from 31 to 92 years with a mean age of 63 years (±10 years); 11 patients were below 50 years. 69 % of patients were male. Table 1 gives an overview of localisation and grade of the OSCC samples.

**Table 1:** Clinicopathological features of included OSCC samples

Localisation		TMN							
	Number (%)	T	Number (%)	N	Number (%)	G	Number (%)	UICC	Number (%)
Mouth floor	23 (27.7)	T1 + T2	56 (67)	N-	53 (64)	G1	2 (2.4)	I	15 (18)
Alveolar ridge	22 (25.4)	T3+ T4	27 (33)	N+	30 (36)	G2	67 (80.7)	II	22 (27)
Tongue	20 (24.0)					G3	14 (16.9)	III	9 (11)
Buccal plain	8 (9.6)							IV	37 (44)
Lip	5 (6.0)								
Palate	3 (3.6)								

### Microarray analysis

Tissue samples used in microarray analysis were composed to 100 % of malignant epithelial cells which was affirmed by a pathologist. The exclusive presence of epithelial cells was also ensured for the control tissue. Expression factors for the candidate genes were analysed according to small and larger tumours (Table 2), to the Union internationale contre le cancer (UICC) classification (Table 3), and to grading (Table 4). Subsequently, one way ANOVA was accomplished and differences in expression were analysed on a level of significance of *p* < 0.05.

**Table 2:** Expression factors of angiogenesis-related genes and IRS score related to T and N in OSCC samples

Gene/Protein	T1 + T2				T3 + T4			
	all (n = 56)	IRS score (n = 10)	N- (n = 39)	N+ (n = 17)	all (n = 27)	IRS score (n = 4)	N- (n = 14)	N+ (n = 13)
VEGF (NM_001025366)	4.13 ± 2.70 (53)	5.8	4.10 ± 2.40 (37)	4.91 ± 4.15 (16)	4.81 ± 2.32 (27)	7.0	5.32 ± 2.68 (14)	4.25 ± 1.72 (13)
PECAM1 (NM_000442)	0.76 ± 0.40 (41)	0	0.55 ± 0.20 (28)	0.58 ± 0.16 (13)	0.52 ± 0.18 (24)	0	0.49 ± 0.21 (13)	0.54 ± 0.14 (11)
EFNB2 (NM_004093)	2.24 ± 1.08 (46)	4.0	2.06 ± 0.90 (32)	2.56 ± 1.25 (14)	2.39 ± 1.12 (24)	5.5	2.4 0 ± 1.04 (13)	2.39 ± 1.22 (11)
ANGPT1 (NM_001146)	0.51 ± 0.32 (51)	1.2	0.41 ± 0.32 (35)	0.33 ± 0.20 (16)	0.29 ± 0.16 (27)	1.5	0.27 ± 0.15 (14)	0.33 ± 0.18 (13)
ANGPT2 (NM_001147)	3.43 ± 1.73 (52)	5.2	3.52 ± 1.73 (35)	3.51 ± 1.08 (17)	3.91 ± 1.92 (25)	5.8	4.40 ± 1.81 (13)	3.39 ± 1.99 (12)

Expression factors were experimentally determined in relation to a healthy oral mucosa pool with an expression factor of 1; displayed is the median; ± means Standard deviation; number of tumour samples in groups are in brackets

**Table 3:** Expression factors of angiogenesis-related genes and IRS score related to UICC in OSCC samples

Gene/Protein	UICC I (n = 15)		UICC II (n = 22)		UICC III (n = 9)		UICC IV (n = 37)	
	all	IRS score (n = 4)	all	IRS score (n = 5)	all	IRS score (n = 2)	all	IRS score (n = 3)
VEGF (NM_001025366)	3.21 ± 1.70 (14)	5.0	4.73 ± 2.59 (22)	6.4	2.89 ± 1.2 (8)	6.0	5.21 ± 3.3 (37)	7.3
PECAM1 (NM_000442)	0.63 ± 0.18 (9)	0.0	0.48 ± 0.17 (18)	0.0	0.48 ± 0.19 (7)	0.0	0.54 ± 0.17 (31)	0.0
EFNB2 (NM_004093)	1.48 ± 0.48 (11)	0.5	2.22 ± 0.93 (22)	6.0	1.60 ± 0.36 (7)	4.0	2.89 ± 1.48 (31)	7.3
ANGPT1 (NM_001146)	0.47 ± 0.18 (12)	1.5	0.31 ± 0.19 (22)	1.2	0.33 ± 0.25 (9)	0.0	0.33 ± 0.17 (36)	2.0
ANGPT2 (NM_001147)	3.81 ± 2.42 (13)	6.5	3.28 ± 1.46 (21)	4.0	2.96 ± 1.12 (9)	6.0	3.77 ± 1.69 (36)	5.7

Expression factors were experimentally determined in relation to a healthy oral mucosa pool with an expression factor of 1; displayed is the median; ± means Standard deviation; number of tumour samples in groups are in brackets

**Table 4:** Expression factors of angiogenesis-related genes and IRS score related to G and N in OSCC samples

Gene/Protein	G1 (n = 2)				G2 (n = 67)				G3 (n = 14)			
	all	IRS score (n = 2)	N- (n = 1)	N+ (n = 1)	all	IRS score (n = 9)	N- (n = 44)	N+ (n = 23)	all	IRS score (n = 3)	N- (n = 8)	N+ (n = 6)
VEGF (NM_001025366)	4.99 ± 3.90 (2)	8	1.09	8.9	4.22 ± 2.46 (65)	5.8	4.64 ± 2.65 (43)	3.47 ± 1.81 (23)	5.62 ± 3.92 (14)	6	3.1 ± 1.25 (8)	8.29 ± 6.44 (6)
PECAM1 (NM_000442)	0.98 ± 0.11 (2)	0	0.87	1.09	0.57 ± 0.18 (54)	0	0.55 ± 0.18 (36)	0.59 ± 0.16 (18)	0.36 ± 0.15 (12)	0	0.24 ± 0.09 (5)	0.47 ± 0.09 (6)
EFNB2 (NM_004093)	1.08 ± 0.69 (2)	4	0.41	1.76	2.36 ± 0.66 (55)	6.7	2.17 ± 0.92 (38)	2.72 ± 1.66 (19)	2.69 ± 1.08 (12)	1.3	2.13 ± 0.9 (5)	3.16 ± 1.18 (6)
ANGPT1 (NM_001146)	1.13 ± 0.17 (2)	6	1.3	0.96	0.34 ± 0.19 (64)	0.7	0.33 ± 0.19 (41)	0.36 ± 0.19 (23)	0.28 ± 0.17 (14)	0	0.36 ± 0.25 (8)	0.2 ± 0.07 (6)
ANGPT2 (NM_001147)	2.55 ± 1.68 (2)	4.5	0.87	4.23	3.53 ± 1.65 (64)	5.9	3.86 ± 1.89 (41)	2.94 ± 1.20 (23)	3.39 ± 1.82 (13)	6.7	1.81 ± 0.45 (6)	5.25 ± 1.47 (6)

Expression factors were experimentally determined in relation to a healthy oral mucosa pool with an expression factor of 1; displayed is the median; ± means Standard deviation; number of tumour samples in groups are in brackets

EFNB2, ANGPT2, and VEGF were overexpressed in 84 % to 98 % of the tumour samples with an average expression factor of 2.3 to 4.5 (Table 2). Expression factors of these genes increase with tumour size and grading (Tables 2 and 4). In subdivision of the UICC classification similar results could be observed, with an unanticipated shift in classification III (Table 3).

In contrast, PECAM1/CD31 and ANGPT1 were found downregulated in 80 % to 95 % of tumour samples with an average expression factor of 0.64 and 0.4, respectively. The expression of PECAM1/CD31 correlated negatively with tumour size and grading (Tables 1 and 3). A similar expression pattern was observed with regard to ANGPT1. In G1 tumours, the expression rate corresponded to healthy mucosa samples. In G2 and G3, a decrease in the expression rate from 1.13 to 0.28 was observed in 95 to 100 % of samples (Table 4). In UICC classification II–IV expression factors of PECAM1 and ANGPT1 remained constant (Table 3).

VEGF and EFNB2 showed a contrary gene expression in small and larger tumours with lymphatic spread. The expression increase in smaller tumours and decrease in larger tumours in contrast to tumours without lymphatic spread (Table 2). Gene expression of ANGPT2 showed no variance in smaller tumours with and without lymphatic spread.

In contrast, in poorly differentiated tumours a higher gene expression of VEGF, EFNB2 and ANGPT2 was observed in tumours with lymphatic spread. In moderately differentiated tumours, gene expression of VEGF and ANGPT2 were higher in tumours without lymphatic spread; only EBFNB2 showed here a higher gene expression (Table 4).

Expression ratio of ANGPT1 against ANGPT2 in smaller and larger tumour is shown in Table 5. Subsequently, one way ANOVA was accomplished and differences in expression were analysed on a level of significance of *p* < 0.05. The ratio was found to decrease with tumour size whereas in tumours with lymphatic spread the ratio remained stable.

**Table 5:** Gene expression ratio of ANGPT1 to ANGPT2

Ratio	T1 + T2	T3 + T4
Ang1/Ang2 all	0.23 ± 0.32	0.15 ± 0.17
Ang1/Ang2 (N-)	0.25 ± 0.37	0.08 ± 0.08
Ang1/Ang2 (N+)	0.16 ± 0.15	0.22 ± 0.18

Ratio in healthy pool 1.0; ± means Standard deviation

### Immunohistochemistry

**Fig. 1:**
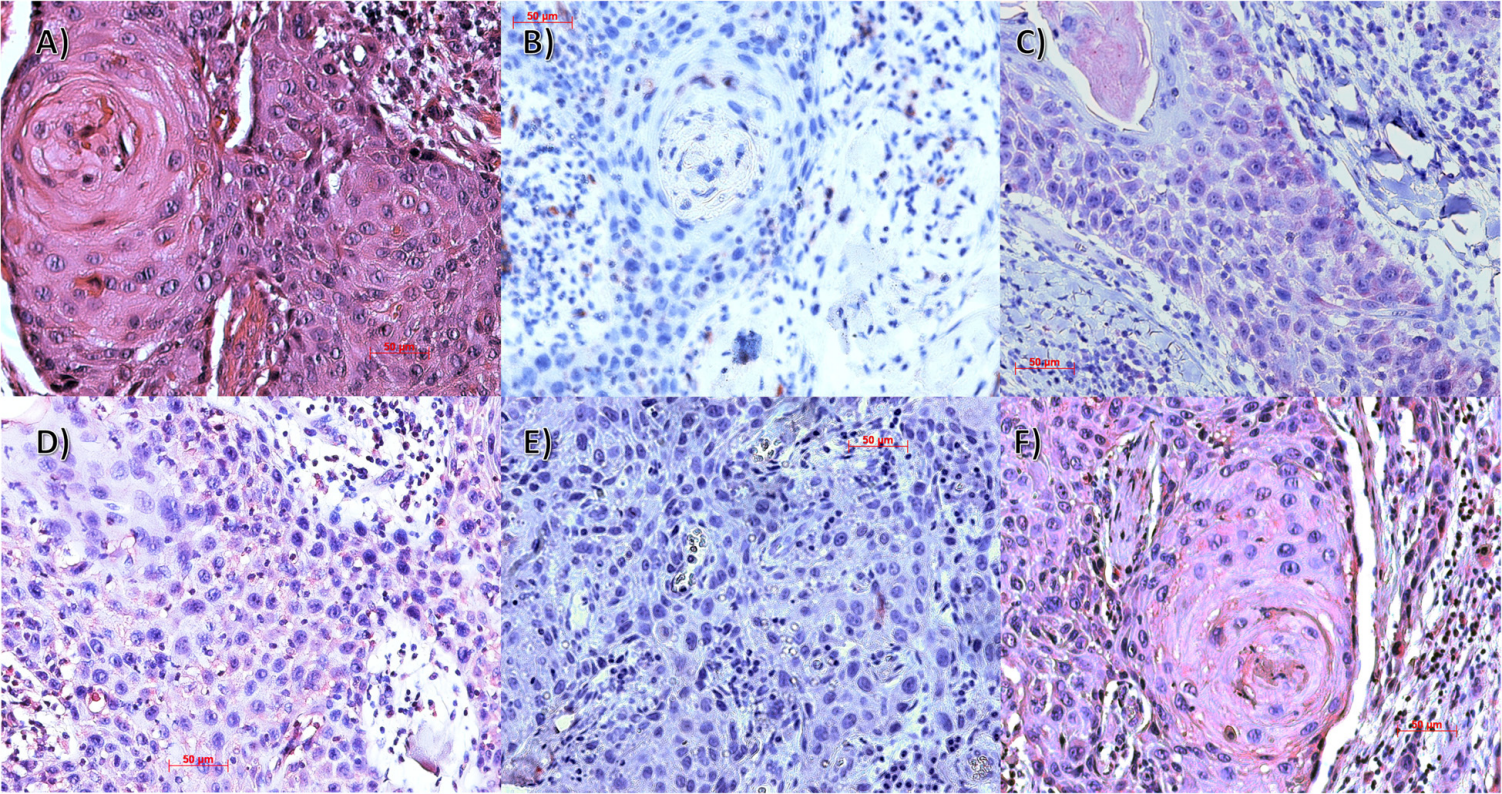
Immunohistochemical detection of CD31 (**b**), Vegf (**c**), Ephrin B2 (**d**), Angiopoietin 1 (**e**) and Angiopoietin 2 (**f**) in a T4 NØ G2 OSCC tumour sample (IHC, x200; HE staining (**a**))

Haematoxylin and eosin staining of tumour specimens was employed to select samples for IHC. Figure 1 gives a review of all staining results using one tumour sample. Tables 2, 3 and 4 present the IRS with reference to classification. Data were subjected to one way ANOVA and differences in expression were analysed on a level of significance of *p* < 0.05. Despite the small number of samples, IRS results were consistent, and validated microarray results.

IHC analysis gave further support of the microarray data: while PECAM1/CD 31 could furthermore not be detected in the analysed samples, the inverse regulation of ANGPT1 and ANGPT2 was also underlined on the protein level. No correlation of gene expression pattern or immunohistochemical detection of VEGF with clinical parameters as well as long time survival or recurrent disease was noticed.

## Discussion

The prognosis of patients suffering from OSCC has been unalterably poor despite much scientific effort to improve the therapy. Tumour-related neo-angiogenesis is an important prerogative for tumour growth and spread; in different tumour entities, e.g. colon carcinoma, anti-angiogenic therapy has proven its effect. Therefore, in this study, we examined the expression of key regulators in the angiogenic and vasculogenic cascade in 83 OSCC tumour samples as the overexpression of single factors during the angiogenic cascade might provide novel points of attack. Our data demonstrate in malignant tumours a significantly higher expression of VEGF, ANGPT2 and EFNB2 at both, gene and protein levels, in malignant tumours when compared to normal oral mucosa. ANGPT1 and PECAM1/CD31 were regulated reversely when compared to ANGPT2. This pattern can be regarded as a characteristic alteration in comparison to healthy tissue. These results are in accordance with findings in the current literature, which describe on the one hand in particular overexpression of ANGPT2 and VEGF, and on the other hand their positive interaction. Li et al. published in 2013 results that underlined the hypothesis that ANGPT2 expression is closely correlated to microvessel sprouting; the vessels themselves however were of reduced maturity and stability [9]. When analysing the overall survival rate they found no correlation between VEGF expression and 5-year survival; nevertheless, this association was discussed controversially in the literature [9].

Further studies have indicated a strong correlation between lymphatic spreading and survival in HNSCC [13, 14]. Jang et al. showed a significant correlation between tumour dimension and biology to lymph node metastases and survival in HNSCC [15]. Remarkable results identified by our data were the 2.7- and 2.9- fold gene expression level of VEGF and ANGPT2 in poorly differentiated tumours with lymph node metastasis versus those without. VEGF and the angiopoietins are described as important actors in anti-angiogenic therapy attacking the microenvironment of the respective tumour [16]. The corresponding expression pattern of VEGF and ANGPT2 hints to a complementary promoting role of both factors in tumour vascularisation even when considering the fact that ANGPT2 has been previously described as an anti-angiogenic mitogen and VEGF antagonist [17]. These observations may provide a possible explanation for the reduced effects of an anti-angiogenic therapy in OSCC; therefore, an additional intervention on the ANGPT2 level might provide a potential therapeutic approach to sensitize target cells to antivascular therapy.

The role of angiopoietins in tumour angiogenesis and progression is not finally defined. Whereas ANGPT2 signalling results in vessel destabilisation, ANGPT1 overexpression is supposed to induce vessel development and maturation [18]. In an IHC investigation of 40 tumour samples, Chien et al. concluded that a strong protein expression of ANGPT1 or ANGPT2 hints to a pronounced biological aggressiveness of the tumour tissue [19]. In our study, we observed no notable overexpression of ANGPT1, neither on the protein nor on the gene level. Thus, ANGPT1 that exerts its effect via Tie-2 signalling seems to play a lower-ranking role as an angiogenic factor in the neo-vascularisation of this tumour entity. In conclusion, the analysis of patient data led us to the suggestion that the progressive lack of ANGPT1 from G1 to G3 tumours comes with reduced vessel ripening which is marked immunohistochemically by weak CD31 staining.

Another striking result elucidated by our analysis of OSCC tumours is the oppositional regulation of ANGPT1and ANGPT2. Evidence in the current literature suggests that the Ang1/ Ang2 ratio can be interpreted as a prognostic factor on gene expression as well as the protein level, whereby a lower ratio is associated with poor prognosis. Li et al. analysed the co-downregulation of ANGPT1 and ANGPT2 protein expression in over 60 tumour samples and concluded from their results that the examined descend of the Ang1/ Ang2 ratio correlates with pronounced vascularisation and poor prognosis [19]. In the presented data, a low ANGPT1/ ANGPT2 expression ratio was observed especially on gene expression level in patients which were diagnosed with a larger tumour size. The ratio in patients with a lymphatic spread stayed unaltered low, however. Particularly in recurrent disease, protein expression of angiopoietins has been scrutinized [20]. Therefore, we conclude that deregulated angiopoietins expression can be linked with a significantly more pronounced biological aggressiveness of the tumour tissue; in particular, the expression of ANGPT1 was associated with lymphatic metastasis. Our findings support the hypothesis that functional vascularity provides a crucial prerogative for the systemic spread of tumour that requires angiopoietin signalling as an initial spark. Hence, analysis of the angiopoietin ratio might emerge as a convincing prognostic factor as the consideration of individual tumour biology could support to plan therapeutic options. It might help to determine the extent of neck dissection or adjuvant therapy. Moreover ANGPT2 seems to play an outstanding role in the development of metastatic spread and thus must be considered as a prospective therapeutic target for attacking progressive disease. Nevertheless, critical questions remain in which point of the signalling cascade the positive effects of a medical intervention outweigh the risk of the induction of malignant transformation when interfering with immune cell interaction [21–23].

EFNB2 seems to be a relevant mediator not only in vessel differentiation but also in vascular sprouting; however, the molecular regulation and downstream signalling as well as the potential role as therapeutic target remain unclear so far. Our analysis here, demonstrated that the upregulated transcriptional regulation of EFNB2 corresponded well with the detected overexpression of VEGF and ANGPT2. Gene expression of EFNB2 correlated with tumour size and tumour differentiation. Also a stronger overexpression was observed in tumours with lymphatic spread against those without. Respectively, Abéngozar et al. were able to inhibit tumour growth as well as angio- and lymphangiogenesis; through the application of highly specific anti-Ephr B2 antibodies EFNB2 was identified as a highly effective antiangiogenic target in a mouse model for the first time [12]. In this model, antibody treatment led to impairment of endothelial cells’ velocity and orientation permanently.

## Conclusion

By analysing 83 tumour samples of OSCC patients, we present here a characteristic expression profile of angiogenic markers that are deregulated on the genetic level. Furthermore, in a subgroup of these markers we confirmed these results additionally on the protein level. Two major findings of our investigation are on the one hand the distinctive expression pattern of the angiogenic mitogens and on the other hand the characteristic inversely correlated Ang1/ Ang2 expression ratio. Starting point for future investigations would be the analysis of vessel maturation in malignant tissue as well as the relevance and reliability of the angiopoietin ratio as a prediction factor of tumour progression and prognosis.
